# Monthly Summary

**Published:** 1882-01

**Authors:** 


### MONTHLY SUMMARY.

*Artificial Milk.*—Although milk is not a mere emulsion of fat and alkali, inasmuch as it contains albuminoids, salts, and sugar, Schischkoff nevertheless found that he could prepare emulsions very similar to milk by closely imitating its natural components; *i. e.*, by first preparing a liquid similar to the more fluid part of milk, and then mixing with it fats of analogous composition to those in real milk. As other chemists had done before him, he found in whey a third albuminoid substance different from either Albumen or Caseine; and he observed in his experiments that while Caseine without Albumen would not yield cream but only milk, and that while the two albuminoids together gave both milk and cream, the presence of the third new albuminoid was necessary in order that the cream should be in a condition similar to natural cream. Schischkoff describes a "good" emulsion as one that smears glass strongly, rises slowly, and forms a decidedly deep layer on standing than would be formed by the fat which is contained in it; it should be white and lustrous. Under the microscope it appears to be composed of small globules of tolerably equal size. It is seen plainly that the act of emulsifying consists in dividing the fat into little globules, and fixing these globules in position by the adhesion of the emulsifying liquid to their surfaces. It was noticed that the globules in the emulsions were apt to be smaller in proportion as the molecular attraction of the liquid for any one of the constituents of the fat was greater, and that in proportion as the globules were smaller the emulsions were less permanent. Shaking promotes the sub-division of the globules, and may consequently hasten the destruction of an emulsion. By long continued shaking the emulsions were, in fact, completely decomposed into fat and soap, which would not again act on one another. The emulsions were destroyed also by cooling, by diluting them with much water, by allowing them to stand for a sufficient length of time, and by manipulations which promote the formation or separation of soap, such as warming or the addition of strong lye, or the like.—*Prof. F. H. Storer, in American Agriculturist.*

---

*A Two Thousand Dollar Tooth.*—A man in a large active business, in New York, said in our hearing: "The worst oversight of my younger days was that somebody did not instruct me to take care of my teeth. At fifty years of age I have but eight natural teeth left, and I could well afford to pay even $2,000 apiece to get back half a dozen or more that I needlessly lost." In explanation he put it in this way: "Artificial teeth are at best a very poor substitute. I am in a large business that needs a good deal of strength of body and mind. All strength comes from good food well digested. But perfect digestion only takes place when food is thoroughly masticated (chewed) and mixed with the saliva, and good, firm, natural teeth are essential for this. So, if I had better teeth I could do a great deal more of profitable business, and earn additional money enough to pay a great price for several of them."

This is worth thinking of by the young. Here are some good rules: 1st. Never crack nuts with the teeth, or bite very hard substances; it breaks or cracks the enamel and hastens decay. 2d. Always brush the teeth before going to bed, if not in the morning also, and use a wooden or quill tooth-pick (not pins or other metal,) to remove any food from between the teeth. If left there overnight it ferments and injures the teeth. Use only a moderately stiff tooth-brush; a very stiff one injures the gums, and promotes decay. Do not use any of the "boughten" tooth-powders, unless it be finely powdered orris root. The most active tooth-powders, which whiten the teeth quickly, contain injurious acids or alkalies. Charcoal, however fine, is not good; it has the "grit" and wear of diamond dust. 4th. If the slightest decay begins on any tooth have a reliable, skillful dentist plug it firmly at once. It will be one of the best possible investments of a small sum for the future.—*American Agriculturist.*

---

*New Treatment of Abscesses.*—Dr. Steven Smith, of Chicago, has inaugurated a new treatment of abscesses, which he affirms to be very successful. It is thus described in the *Chicago Medical Review*: "When the abscess points it is opened and the contents evacuated. The cavity is then injected with carbolised water, and over-distended for two or three minutes. The water is then pressed out, and over the whole area undermined by the cavity, small, dry, compressed sponges are laid and bound down with a bandage. Carbolised water is then applied to the bandage and injected between its layers until the sponges are thoroughly wet, after which a dry bandage is applied over all. The sponges by their expansion make firm and even compression upon the walls of the abscess, and hold them in perfect apposition, thus favoring a union. The dressing is left on for five or six days, unless there is a constitutional disturbance, or pain in the seat of the former abscess. It is found, in most cases, when the bandage is removed, that the abscess has completely closed by an approximation of its walls, and the external wound heals readily under a simple dressing of carbolised oil. A case was recently seen where this admirable result was secured in a child, although the abscess was a large one, originating in caries of the head of the femur, and opening on the outside of the thigh. No constitutional disturbance, no discharge, no re-accumulation, and no pain followed its use. Mammary and submammary abscesses have been treated by this method with excellent results."

---

*About Raising Healthy Women.*—When there is a lack of warm clothing there is always a drain upon the vital energies which tends to break down the health, and this sometimes takes place when the victim has such a good constitution and such excellent health, as to be able to bear with impunity almost any neglect or exposure. I know of women who wear thin cotton stockings all winter, and who say that they do not need anything warmer in-doors, but these are not healthy women, and I doubt if their supposed "feelings" correctly indicate the body's needs. Those who dislike the feeling of wool in contact with the skin (and I think that this is much according to habit or use,) can wear a thin pair of cotton stockings under woollen ones, which is better for warmth than two pairs of cotton stockings.

Nourishing food, pure air, plentiful sleep, and happy exercise, are all essential to perfect health. Nutritious food is necessary for good blood, and this cannot be pure, unless clean, fresh air be bountifully supplied. Growth and repair cannot go on properly without the entire rest of healthy, unforced sleep, and exercise of both body and brain is necessary to the health and strength of each.—*American Agriculturist.*

---

*A Third Set of Teeth.*—Mrs. Elizabeth Sutphin, an aged lady, who resides in New Brunswick, N. J., and who is now in her 93d year, is cutting her third set of teeth, five of which have already shown themselves through the gums. The process is accompanied with almost all the symptoms usually observed in a child. Mrs. Sutphin, who is a sister of Mr. Wm. Rhodes, (who is 83 years of age,) is a sprightly lady, considering her extreme age. Her mind is perfectly clear and her memory remarkable. She will relate events that happened years ago, giving the date thereof with exact accuracy.

---

*Largest Book Published.*—The new edition of Webster's Unabridged Dictionary, just issued, is believed to be, in the quantity of matter it contains, by far the largest volume published. It now contains about 118,000 words defined, and nearly 15,000 words and meanings not found in any other dictionary. The Biographical Dictionary, just added, supplies a want long felt by the reader and student, in giving the desired information so briefly. Never was any one volume so complete as an aid in getting an education.

